# The Roles of Angiogenesis in Malignant Melanoma: Trends in Basic Science Research over the Last 100 Years

**DOI:** 10.5402/2012/546927

**Published:** 2012-06-07

**Authors:** D. Dewing, M. Emmett, R. Pritchard Jones

**Affiliations:** ^1^Department of Molecular and Clinical Cancer Medicine, Mersey Academic Plastic Surgery Group, Liverpool Cancer Research UK Centre, The Duncan Building, Daulby Street, Liverpool L69 3GA, UK; ^2^Department of Plastic Surgery, Whiston Hospital, Warrington Road, Prescott, Merseyside L35 5DR, UK

## Abstract

Blood vessels arose during evolution carrying oxygen and nutrients to distant organs via complex networks of blood vessels penetrating organs and tissues. Mammalian cells require oxygen and nutrients for survival, of which oxygen has a diffusion limit of 100 to 200 **μ**m between cell and blood vessel. For growth beyond this margin, cells must recruit new blood vessels, first by vasculogenesis, where embryonic vessels form from endothelial precursors, then angiogenesis which is the sprouting of interstitial tissue columns into the lumen of preexisting blood vessels. Angiogenesis occurs in many inflammatory diseases and in many malignant disease states, including over 90% of solid tumours. Malignant melanoma (MM) is the most lethal skin cancer, highly angiogenic, highly metastatic, and refractory to all treatments. Raised serum levels of vascular endothelial growth factor (VEGF) strongly correlate MM disease progression and poor prognosis. Melanoma cells secrete several proangiogenic cytokines including VEGF-A, fibroblast growth factor (FGF-2), platelet growth factor (PGF-1), interleukin-8 (IL-8), and transforming growth factor (TGF-1) that modulate the angiogenic switch, changing expression levels during tumour transition from radial to invasive vertical and then metastatic growth. We highlight modern and historical lines of research and development that are driving this exciting area of research currently.

## 1. Angiogenesis

Angiogenesis was first associated with malignancy 100 years ago [1] being new vessel growth from a pre-existing blood supply. It is physiologically tightly regulated during wound healing, embryogenesis, and female reproduction. It also occurs in several pathological conditions including malignant melanoma. In 1919, Krogh described a tissue cylinder surrounding an axial blood vessel allowing oxygen and glucose diffusion for metabolism [2]. In 1945, vascularised tumour cell transplants used *in vivo* murine models survived to promote tumour survival and growth [3]. A diffusible “angiogenic” substance was later proposed in 1968 using a hamster cheek pouch model demonstrating choriocarcinoma vasoproliferation [4]. In 1971, Folkman postulated that tumour growth and metastasis driven by angiogenesis might be blocked by inhibiting angiogenesis (6). Later it was recognised, ischaemic stress occurs as the tumour growth exceeds the distances of Krogh's cylinder, resulting in ischaemic necrosis, or ischaemic/hypoxic induced activation of angiogenesis [5] with diffusion limits of oxygen for cell survival measured at 100–200 microns [6]. Beyond this margin, angiogenesis facilitates cell growth and survival, demonstrated experimentally with cultured tumor cells in avascular rabbit cornea attracting new capillaries and vascularizing the expanding tumor [7]. In 1976, Gullino showed precancerous cells acquiring angiogenic capacity in a sequence leading to cancer [8], leading to a concept of “angiogenic switch” [9]. This is postulated to be crucial to angiogenesis with the switch “off” when pro-angiogenic molecules are balanced by anti-angiogenic molecules, and “on” when this balance is reversed [10, 11]. “Switch” triggers include low pO_2_, low pH [12] or hyper/hypoglycaemia or hyperthermia [13], mechanical stress, immune/inflammatory response, and genetic mutations [14, 15].

## 2. Vascular Endothelial Growth Factor (VEGF)

Central to angiogenesis is VEGF, first isolated in 1989 [16]. VEGF promotes endothelial cell proliferation, survival, migration, vasodilatation, and vasculogenesis by recruiting bone marrow-derived haematopoetic progenitor cells [17, 18]. VEGF is a heparin-binding family of glycoproteins including VEGF-A, VEGF-B, VEGF-C, and VEGF-D. VEGF-A occurs in at least four isoforms of 121, 165, 189, and 201 amino acids length, because of alternative gene splicing. VEGF-A commonly referred to as VEGF is overexpressed in almost all solid tumours and correlates with vascularity, grade, and prognosis [19]. It is also expressed by dendritic and macrophage immune cells infiltrating into tumour stroma [20]. VEGF ligands bind with variable affinity to tyrosine kinase receptors expressed on blood endothelial cell surfaces with vascular endothelial growth factor receptors (VEGFR) VEGFR-1 and VEGFR-2 involved in angiogenesis by their binding of VEGF-A isoforms. VEGFR-3 is expressed on lymphatic endothelial cells and is involved in lymphangiogenesis, binding VEGF-C and VEGF-D.

## 3. The Role of VEGF in Melanoma Angiogenesis

Neovascularisation's importance in human cutaneous melanomas was demonstrated to indicate angiogenic activity [21] and VEGF's role in melanoma angiogenesis was first demonstrated with the successful transplantation of human melanoma fragments into a hamster cheek pouch [22]. Tumor blood flow in melanomas thicker than 0.9 mm was detected using Doppler ultrasound [23], and endogenous VEGF expression and secretion in melanoma tumour cells were later established [24]. Murine studies have examined several aspects of VEGF expression and its role in tumour growth. Transfection and overexpression of VEGF isoforms in cell lines normally producing baseline VEGF levels have been an invaluable tool for identifying differences in tumorigenicity between isoforms. VEGF_121_ and VEGF_165_ promote aggressive tumour growth in mouse xenografts, contrasting VEGF_189_ (high heparin affinity/lower bioavailability) where overexpression demonstrates poor tumour growth [25].

*In vivo* murine studies have also shown that aggressive melanoma cell lines express higher levels of VEGF compared to nonaggressive cell lines [26]. Nonaggressive cell lines such as Mel-2 transfected to overexpress VEGF, demonstrated conversion to an aggressive phenotype producing large vascularised nonnecrotic tumours in mouse models. These effects could be reversed with antisense VEGF transfection resulting in small poorly vascular tumours [27]. These findings demonstrate VEGF's role in aggressive tumour behaviour.

VEGF-A isoform behaviour may vary with environment. Nonmetastatic skin melanoma (SKMEL) cells transfected to overexpress murine VEGF_164_, an equivalent to human VEGF_165_, were subcutaneously implanted into mice, and demonstrated neovascularisation [27]. Brain metastatic cells from the human melanoma cell line Mel57 were transfected to overexpress VEGF_165_, and coopted pre-existing intra- and peritumoural vessels without inducing neovascularisation [28]. Are these clues to MM resistance to treatments, with tumour behaviour varying according to environment?

Surprisingly VEGF is difficult to detect in skin [29] and is localised in dermal endothelium but not epithelial keratinocytes [30] or benign naevi. Dysplastic melanocytes produce FGF-2 and VEGF. MM by comparison to normal melanocytes, greatly overexpresses bFGF thereby stimulating endothelial cell growth and further production of VEGF [31]. Significantly an increase in the secretion and stromal deposition of VEGF is demonstrable during the switch from radial to vertical growth of MM [32] evidencing a role for VEGF in the “switch” mechanism. Clinical investigations with VEGF give conflicting conclusions.

Immunohistochemical studies demonstrated upregulation of VEGF_165_ and VEGF_121_ and increased microvascular density in primary melanomas, strongly correlating disease progression [30, 32, 33]. Conversely a similar analysis showed only tumour thickness as an independent variable associated with disease-free survival (Breslow classification) and overall survival—predicted by depth of tumor infiltration (Clark classification). Another study found increased vascularity actually correlated with survival [34].

Bridging this gap in understanding may be the discovery of anti-angiogenic VEGF isoforms which until recently could not be isolated from their pro-angiogenic sister isoforms. These differ from pro-angiogenic isoforms due to gene splicing in the 8th exon of the VEGF gene, resulting in same length final protein product, but with a different terminal base sequence encoded in the 8th exon due to splicing at a novel distal splicing site, conferring anti-angiogenic properties, denoted VEGF_xxx_b [35].

*In vitro* and *in vivo* evidence show VEGF_xxx_b isoforms competitively bind VEGFR-2 with equal affinity to pro-angiogenic isoforms, preventing phosphorylation and down-stream intracellular signaling of angiogenic processes [36]. *In vivo* experiments with VEGF_165_b transfected melanoma cells in nude mice demonstrated reduced tumour growth and vessel density [37]. Studies with melanoma samples demonstrated VEGF_xxx_b to be constitutively expressed in normal surrounding epidermis, but reduced in primary melanoma samples (both horizontal and vertical growth phases) which developed metastasis irrespective of primary tumour thickness. Total VEGF expression, however, demonstrated staining in metastatic and Nonmetastatic melanomas and normal epidermis. Diminished VEGF_xxx_b expression may predict metastatic spread in patients with primary melanoma being crucial in pushing the angiogenic switch, due to imbalanced VEGF gene splicing to pro-angiogenic isoforms [38].

Overall, these experimental endeavors have translated into modest clinical gains, the benefits of which are still being evaluated and developed. VEGF serum levels do show prognostic utility, confirming clinically angiogenesis' importance in MM [39] and a role is being developed for therapy response monitoring in clinical trials.

## 4. Angiopoietins and the TIE Receptor Axis

Angiopoietins bind the receptor tyrosine kinase (RTK) family of Tie-1 and Tie-2 receptors and are required for embryonic and adult angiogenesis, interacting with VEGF in the regulation of angiogenesis and tumour growth through RTK receptor activation [40]. Experimentally Ang-1 agonism of Tie-2 promotes vascular maturation through pericyte mediation maintaining endothelial cell (EC) quiescence, cell survival in pericytes treated *in vivo* with TNF-*α* (whilst pericytes treated with Ang-2 undergo apoptosis), and pericyte migration [41]. In human umbilical vascular endothelial cells (HUVECs), VEGF induces Tie-2 and may therefore promote angiogenesis by inhibition of EC vascular stabilization [42]. Raised serum levels of Ang-2 are also associated with advanced disease in melanoma offering a potential biomarker for disease monitoring.

## 5. Interleukin-8 (IL-8)

IL-8 chemokines are synthesized by macrophages and endothelial cells primarily inducting chemotaxis in target cells. Benign melanocytic lesions and normal epidermis express minimal levels; however, in MM patients IL-8 serum levels are significantly higher and correlate with advanced disease stage and overall patient survival [43]. IL-8 expression in melanoma cells is upregulated in response to TGF-1, and is pro-angiogenic in mouse xenograft models. Tumour production of IL-8 not only drives melanoma cell growth but also promotes tumour cell migration, whilst endothelial IL-8 induces endothelial cell migration [44].

## 6. Platelet-Derived Growth Factor (PDGF) and PDGFR**β** Axis

Pericyte and EC crosstalk is influenced by platelet derived growth factor (PDGF) which has 5 isoforms, and its TK receptor platelet derived growth factor receptor (PDGFR) PDGFR*β*. Activation by PDGF leads to receptor dimerisation, autophosphorylation, and signaling transduction through the PI3K pathway important in EC migration [45]. During angiogenesis this axis promotes recruitment of pericytes to the unstable neoangiogenic vasculature. Pericytes are relatively undifferentiated cells normally lining the outer surface of endothelial cells contributing to stability and angiogenesis by producing VEGF. Overexpressing PDGF-BB or PDGF-DD isoforms in a Nonmetastatic B12 mouse melanoma cell line demonstrated paracrine effects on tumour growth with pericyte recruitment and coverage of tumour vessel. The observed reduction in apoptosis and increased tumour growth is suggested to be due to increased pericyte expression of VEGF [46]. Endothelial progenitor cells (EPCs) subjected to hypoxic conditioning demonstrate higher levels of PDGF-BB isoform compared to cells in normoxic conditions exerting positive effects on HUVEC cells including increased proliferation and migration, not observed in HUVECs exposed to recombinant PDGF-BB alone [47]. These models examining the effects of hypoxic conditioning on PDGF overexpression on endothelial cell behaviour suggest an important role for the PDGF/PDGFR signaling axis in future therapeutic approaches and an important pathway through which hypoxia stimulates angiogenic behaviour.

## 7. Placental Growth Factor (PGF)

PGF isoforms result from gene splicing and exhibit variable heparin-binding. PGF-1 and -2, are expressed by melanoma cells binding neuropilin receptors NP-1 and -2 expressed on ECs. NPs are VEGF coreceptors involved in axon guidance and cell survival and migration [48]. PGF may also influence MM development by binding VEGFR-1 receptors on haematopoietic bone marrow precursors inducing mobilization and recruitment of inflammatory mediators that upregulate VEGF production around the tumour, and by binding VEGFR-1 expressing smooth muscle cells and pericytes, thereby affecting blood vessel maturation and stability [49]. Tumour vessels commonly lack functional pericytes, which are normally protective against changes in oxygen or hormonal balance by physically stabilising the vasculature. The effect of this is to allow hypoxic stimulation of tumour angiogenesis to go unchecked by the loss of vasoactive control [50]. PGF also binds VEGF-A forming heterodimers, directly enhancing melanoma angiogenesis by VEGFR-2 activation of ECs [51].

## 8. Extracellular Matrix (ECM)

The ECM is a complex environment for biochemical and cellular regulation of gene expression, differentiation, adhesion, and migration supporting cells and related structures [52]. EC migration into tumour stroma is essential for metastasis, and ECs and tumour cells increase metallo-matrix proteinase (MMP) expression to facilitate this process [53]. The gelatinases MMP-2 and MMP-9 degrade basement membrane and were the first MMP's linked with angiogenesis [54] correlating with invasive and metastatic phenotypes [55]. MM expresses several MMPs including tissue inhibitor metalloproteinases (TIMPs) [55]. Raised serum levels of MT1-MMP and MMP-9 are strongly associated with rapid disease progression. Raised serum MMP-9 expressed exclusively in the horizontal phase of melanoma growth is strongly associated with bone metastases. By contrast, nondysplastic melanocytes express basal levels of MMP-1 and -9 [56]. Angiogenic mitogens, such as bFGF and VEGF, play a key role in stimulating capillary endothelial cells to produce MMPs [57]. Studies also demonstrated MMPs involvement in the angiogenic switch. A tumour model reliably replicating this switch demonstrated MMP-2′s important role in developing an angiogenic phenotype [58]. Another demonstrated MMP-9 influencing the angiogenic switch in a pancreatic tumor model [59]. These findings suggest MMP/VEGF interaction is critical in initiating angiogenesis and promoting tumour invasiveness.

## 9. Basement Membrane

Basement membrane (BM) is specialised ECM separating ECs from underlying mesenchyme [60]. BM comprises collagen type IV, laminin and fibronectin, and heparan sulphate proteoglycans (HSPG) [61]. Heparanase secreted from melanoma cells cleaves heparan sulphate (HS), degrading this barrier that is normally protective of the basement membrane's underlying type IV collagen structure from proteolysis. This exposes a ligand-integrin binding site for angiogenesis not found in quiescent vessels [62]. Heparanase also liberates and activates heparin-binding growth factors bFGF and VEGF from the ECM, which may act in a paracrine manner with the tumour [63]. Several melanoma cell lines degrade ECM through heparanase enzyme degradation [64] and pathological specimens of melanoma tissue demonstrate increased levels of heparanase mRNA at all stages of tumour development, with highest expression found in vertical growth phase samples [65]. Transfection of the heparanase gene into nonaggressive MM cell lines demonstrates significantly increased enzyme activity and invasiveness [65]. Heparan sulphate break-down products also suppress T-lymphocyte immune surveillance in the environment around the tumour [66]. It is also hypothesized that invasive melanoma cells may arise by a macrophage-transformed melanocyte fusion, allowing the tumour to acquire a “macrophage phenotype,” improving tumour migratory potential and allowing it to bypass immune surveillance mechanisms [67–69].

## 10. Hypoxia-Driven Pathological Angiogenesis

Hypoxia strongly stimulates angiogenesis physiologically or pathologically, and oxygen tension is a key regulator of VEGF gene expression *in vivo* and *in vitro* [70], with VEGF mRNA expression induced by low oxygen concentrations in normal or transformed cultured cells [71]. In melanoma, hypoxia independently upregulates VEGF [72] and tissue factor (TF) production [73]. TF is a factor VII receptor and a pathway of hypoxic stimulation for VEGF triggering the coagulation cascade. In melanoma, this potentiates tumour cell binding, with cytoplasmic tail tumour cells required for VEGF synthesis [74]. The second pathway for hypoxic stimulation is through hypoxia inducible factor (HIF) *α* “master switch” for transcriptional regulation of cellular responses to hypoxia, controlling diverse target genes including glycolytic metabolism, erythropoiesis, and vascular remodeling. HIF is a 28-base sequence found in the 5′ promoter of the VEGF gene, and mediates hypoxia-induced transcription of VEGF [75]. It is regulated at protein level by the Von Hippel-Lindau protein (VHLP) which controls HIF degradation in normoxic conditions [76]. VHL disease unsurprisingly is strongly associated with angiogenic neoplasms [77].

## 11. Hypoxia and Melanoma

Human epidermis lacks vasculature and is markedly hypoxic. The O_2_ tension (pO_2_) in dermis has a pO_2_ of 10%, whilst in some skin appendages the pO_2_ ranges from mildly (5%) to severely hypoxic (0.5%) [78]. Human skin exhibits extensive binding of hypoxia-sensitive agents (such as nitroimidazole EF5) in the basal epidermal compartment with increased expression levels of the oxygen sensor HIF-1*α* [79] and it is this already hypoxic basal epidermis that may provide a permissive environment for pushing resident dysplastic melanocytes into a pro-angiogenic state with hypoxia pushing the angiogenic switch. Physiologically mild hypoxia of the skin can promote melanocyte transformation [80, 81] and correlate with increased expression of HIF-1*α*, HIF-2*α*, and VEGF, found commonly in melanoma samples, and poor prognosis and survival [82]. Hypoxia directly stimulates tumour blood vessel formation in response to HIF induced VEGF production. In MM, this results in vessels structurally and functionally abnormal with shunts contributing disordered tumor blood flow [83] exacerbating hypoxic/acidic regions in the growing tumour [84]. Mismatched vascularity and oxygenation further influences the balance of angiogenic stimulators and inhibitors in favor of angiogenesis, aiding malignant/metastatic cell selection [85].

## 12. Conclusions

Angiogenesis is a hallmark of cancer [10], and VEGF is a key player [16]. Evidence shows its involvement at key elements of tumour growth; including tumour hypoxia triggering HIF-induced VEGF production, endogenous tumour production of VEGF, tumour-endothelium paracrine interaction and upregulation of VEGF, liberation of VEGF from the ECM by tumour heparanase production, or recruitment of VEGF producing macrophages. Whether these processes are sequentially linked or not, the common denominator is VEGF. Anti-angiogenic isoforms are gradually emerging in the research sphere [35] and may provide invaluable prognostic information [38], and in the future therapeutic possibilities. We hypothesise that melanoma cells express at low levels (unpublished data) VEGF receptors in pro- or anti-angiogenic isoforms, the balance of which is governed by splicing, in turn influenced by factors that modulate the angiogenic switch, and upregulating VEGF receptor expression as a tumour survival response. This process of “survival” growth may facilitate further tumour growth by causing localised intratumoral hypoxia which is a powerful stimulus to HIF-1*α* production and therefore more neovascularisation. Upregulating VEGF receptors at the tumour surface or tumour-endothelial cell interface may occur as a prelude to tumour ECM interaction with MMPs. Whether the angiogenic switch is pushed by a preponderance of proangiogenic VEGF or by VEGF splicing from anti- to pro-angiogenic forms is unclear; however, this critical event appears directly related to the tumour change from radial to invasive growth and may represent the culmination of a sequence of processes. This relationship has not yet been linked in a unified model that gradually upregulates pro-angiogenic VEGF to this critical level.

Melanoma cells also produce heparanase disrupting the basement membrane facilitating tumour cell invasion. Macrophages possibly add fuel to the fire bringing greater quantities of VEGF to the tumour site setting up new VEGF gradients favoring tumour growth, which in turn may upregulate MMPs and therefore metastatic behaviour. Heparin-treated tumour cells demonstrate reduced cell adhesion and migration [86], and given that heparin-binding sites are spliced during synthesis of VEGF isoforms, investigating how heparin may affect the efficacy of VEGF isoforms on tumour growth *in vitro* would be intriguing.

A tumour thus enriched by numerous sources of pro-angiogenic growth factor, and at the same time appearing to enjoy immune privilege, is able to liberate VEGF from the ECM which acts as a standing reservoir of VEGF and facilitate invasion and metastasis.

Future research might focus on tumour VEGF isoform characterization, distribution and site-specific variability, and the influence on disease progression. Examining the possible role of splice variants in hypoxic states such as pregnancy in melanoma (indeed murine models show increased lymphangiogenesis and metastasis in pregnancy [87]) could yield valuable insights.
